# The Hajj legacy and Saudi Arabia’s exemplary response to COVID-19

**DOI:** 10.3389/fpubh.2025.1520179

**Published:** 2025-06-02

**Authors:** Ghadah Alsaleh, Bander Balkhi, Ahmed Alahmari, Anas Khan

**Affiliations:** ^1^Global Center of Mass Gatherings Medicine, Ministry of Health, Riyadh, Saudi Arabia; ^2^Department of Clinical Pharmacy, College of Pharmacy, King Saud University, Riyadh, Saudi Arabia; ^3^King Saud University, Riyadh, Saudi Arabia

**Keywords:** pandemics, public health, COVID-19, mass gathering, Saudi Arabia

## Abstract

The COVID-19 pandemic required strong public health measures globally. Saudi Arabia’s effective pandemic management, leveraging its experience with mass gatherings such as the Hajj pilgrimage, has been lauded globally. This study was developed using a narrative synthesis approach, based on a structured review of peer-reviewed literature (PubMed and Scopus) and official sources (Saudi MoH and the WHO) covering March 2020–December 2024. This study examines Saudi Arabia’s response to the COVID-19 pandemic, with particular emphasis on the strategies implemented to safeguard the Hajj pilgrimage. The analysis is framed within the context of the World Health Organization’s (WHO) COVID-19 After Action Review pillars, providing a structured evaluation of the Kingdom’s efforts to mitigate risks and protect both pilgrims and the broader population. Topics covered include country-level coordination, risk communication, surveillance, border health, national laboratory systems, infection prevention, case management, operational support, and essential health services. Findings show that preexisting infrastructure and mass-gathering expertise enabled rapid activation of multisectoral task forces, adaptive risk-communication campaigns, and scalable testing and isolation protocols. The Hajj legacy strengthened laboratory diagnostics and surge staffing, informed border screening algorithms, and guided large-event risk assessments. Integrating mass-gathering experience with WHO’s framework fostered resilience to complex health emergencies. Saudi Arabia’s model offers actionable insights for other nations seeking to harness cultural and organizational strengths in pandemic preparedness.

## 1 Introduction

The emergence of the COVID-19 pandemic prompted a global effort to develop preparedness strategies and response plans to understand and contain the novel virus (1). As a nation with a population of approximately 34 million, Saudi Arabia found itself at the forefront of this global challenge (2). With a robust healthcare system comprising over 494 hospitals and 75,225 hospital beds, Saudi Arabia was well-positioned to address the pandemic (3).

The Kingdom of Saudi Arabia was one of the first countries to take precautionary and preventive measures to address the COVID-19 pandemic, both domestically and internationally, recognizing its global impact. Leveraging its experience in risk management and health security from previous incidents such as MERS-CoV, the Kingdom implemented early precautionary measures to secure national and global health, ensure the security and the safety of Hajj and Umrah pilgrims every year, and develop a highly efficient health system. These included establishing Command and Control Centers, halting travel to China, and suspending tourist visas. As COVID-19 cases emerged, the Kingdom enforced social distancing, suspended activities such as Umrah and Hajj and education, and conducted mass testing. Additionally, the Kingdom established guidelines in line with the standards adopted from the WHO’s document titled “Operational Planning Guidelines to Support Country Preparedness and Response” to enhance its response to the pandemic in terms of preparedness, detection, testing, tracing, isolation, and treatment. These guidelines served as a comprehensive framework supplementing the proactive measures already in place. Together, these actions formed an integrated national strategy aimed at mitigating the impact of the pandemic and ensuring the safety and wellbeing of its citizens and residents, while also contributing to global health initiatives as a reference point for effective response measures (4).

The annual Hajj pilgrimage, which attracts millions of Muslims from around the world, presented a particular concern due to the potential for rapid disease transmission in such a large gathering. A mass gathering event, as defined by the WHO, refers to “events characterized by the concentration of people at a specific location for a specific purpose over a set period of time and which has the potential to strain the planning and response resources of the country or community.” Thus, ensuring the safety of pilgrims and the broader population became a paramount priority. Since discovering the first COVID-19 case in the Kingdom on 2 March 2020, the number of confirmed cases and their contacts increased in different regions in the country. As of 23 August 2020, the Kingdom recorded 307,479 confirmed COVID-19 cases and 3,649 COVID-19 deaths. In parallel, the country increased diagnostic tests, preventive measures, and preparedness procedures to contain the spread of the disease, resulting in a total of 280,143 recoveries (5). Notably, the Kingdom maintained one of the lowest COVID-19 case fatality rates among G20 nations at 0.9%, while the global case fatality rate stood at 2.2%, emphasizing the effectiveness of its early and decisive actions (6).

In the face of the COVID-19 crisis, Saudi Arabia demonstrated exemplary leadership and commitment to safeguarding the health and wellbeing of both its citizens and the millions of pilgrims who participate in the Hajj each year (5). The Kingdom’s proactive and comprehensive response to the pandemic, driven by its extensive experience in managing large-scale public health events, has been hailed as a model for other countries to follow (6). This study explores Saudi Arabia’s response to the COVID-19 pandemic, with a particular focus on the measures implemented to ensure the safe conduct of the Hajj pilgrimage. This study examines how the insights gained from organizing the Hajj uniquely prepared Saudi Arabia to address the challenges posed by the COVID-19 pandemic. The annual Hajj planning process involves extensive year-long preparations that encompass all levels of engagement: strategic, tactical, and operational.

This study was developed using a narrative synthesis approach, based on a structured review of both peer-reviewed literature and official institutional sources. Relevant academic studies were identified through searches of PubMed and Scopus, while government policies and response strategies were drawn from official publications by the Saudi Ministry of Health, the WHO, and other public agencies. The data reviewed spans from March 2020 to December 2024, providing a comprehensive overview of Saudi Arabia’s pandemic response, particularly in relation to the planning and execution of the Hajj during COVID-19.

## 2 Preparation and planning

Saudi Arabia’s proactive approach to the COVID-19 pandemic was evident in the meticulous planning and preparation undertaken to safeguard public health, particularly during the Hajj pilgrimage, a mass gathering with inherent risks of disease transmission. Saudi Arabia’s experience in managing the complexities of the Hajj proved invaluable in developing a robust response framework.

Saudi Arabia’s stringent measures in 2020, including restricting the pilgrimage to a small number of local pilgrims and implementing rigorous health protocols, resulted in zero reported COVID-19 cases among the participants. This successful initial response highlights the efficacy of Saudi Arabia’s approach to mitigating pandemic risks during the Hajj (5). The following subsections will detail the key components of Saudi Arabia’s preparedness and planning, emphasizing the strategic decisions and actions taken to address the emerging crisis and ensure the safety of both pilgrims and the broader population (7).

### 2.1 Hierarchical structure and chain of command in the response

Saudi Arabia’s response to COVID-19 unfolded in two strategic phases, each designed to maximize the efficiency of the response and leverage the full capacity of key institutions. The first phase was initiated by a Royal Decree, which led to the establishment of the High Committee. This committee consisted of senior officials from critical ministries and agencies, including the Ministry of Health, the Ministry of Finance, the Ministry of Media, the Ministry of Foreign Affairs, Weqaya (Saudi Center for Disease Prevention and Control), the General Authority of Civil Aviation (GACA), and the Ministry of Interior. The inclusion of these diverse institutions was not only essential for addressing the immediate pandemic risks but also reflected Saudi Arabia’s broad institutional capacity in handling such a crisis (8).

The strength of the High Committee lies in its comprehensive and integrated structure. Each entity brought unique expertise to the table, from health surveillance and logistics to media communication and international relations. This multidisciplinary approach was vital in crafting an effective and cohesive response. Saudi Arabia’s extensive experience in managing the Hajj pilgrimage, a mass gathering of millions of people, provided a solid foundation for the High Committee’s ability to implement strict health protocols and coordinate large-scale logistics. The experience of these institutions, coupled with their high operational readiness, made the High Committee uniquely capable of leading the country’s response during the pandemic (Table 1).

**Table 1:** Saudi Arabia’s response to COVID-19.

Category	Key measures and contributions	Impact
Coordination and Planning	Utilized experience from Hajj for agile pandemic response; refined risk management and real-time monitoring.	Enhanced pandemic response agility and coordination.
Risk Communication and Community Engagement	Applied Hajj communication strategies; managed misinformation and infodemics with media campaigns.	Improved public information dissemination and community trust.
Surveillance and Contact Tracing	Used Health Early Warning System (HEWS) and mass screening initiatives.	Effective case identification, isolation, and tracing.
Border Health and Points of Entry	Implemented strict measures like social distancing and quarantine; updated procedures post-vaccine.	Controlled entry of potentially infected travelers; maintained safety.
Infection Prevention and Control	Extended Hajj hygiene protocols; developed mandatory training for healthcare workers.	Maintained high standards of infection control and prevention.
Case Management and Knowledge-Sharing	Repurposed facilities and resources; integrated the latest research and innovations.	Adapted healthcare facilities to manage surges and enhance treatment protocols.
Operational Support and Logistics	Utilized Hajj logistics for the distribution of medical supplies and vaccines.	Ensured efficient supply chain management and resource allocation.
Essential Health Services	Ensured continuity of healthcare services; supported public pharmacies and telehealth.	Minimized disruptions to essential services and patient care.
COVID-19 Vaccinations	Applied Hajj experience for efficient vaccine rollout; digital health transformation for tracking.	Achieved high vaccination rates and controlled virus spread.
Legislation and Financing	Adapted legal and financial frameworks from Hajj for pandemic response; provided free COVID-19 treatment.	Enabled rapid legislative action and financial support.
Public Health and Social Measures	Implemented social distancing, mask mandates, and crowd control; updated protocols regularly.	Controlled virus transmission and maintained public safety.
Mental Health and Psychosocial Support	Integrated psychosocial support strategies from Hajj; provided counseling and community initiatives.	Addressed mental health impacts and supported community well-being.

The High Committee was responsible for formulating and overseeing precautionary measures, including travel restrictions, quarantine protocols, and mass event management. Its agility was supported by a deep understanding of large-scale public health, shaped through years of managing health risks during the Hajj. This experience, along with existing infrastructure for mass gatherings, enabled a rapid and effective response to COVID-19.

Following a comprehensive global assessment, the response transitioned into a second phase with the formation of the Concerned Committee, chaired by the Minister of Health. This phase expanded the scope of involvement to a broader set of entities, reflecting the need for a sustained, multisectoral approach. The shift marked a move from emergency response to continuous adaptation, with a clear command structure and strong cross-sector coordination ensuring the effectiveness and flexibility of Saudi Arabia’s evolving response.

### 2.2 Country-level coordination, planning, and monitoring

The meticulous planning, prepared through years of organizing the Hajj, provided the groundwork for the country’s agile and comprehensive pandemic response strategy. The ability to monitor and adapt plans in real-time reflects the nation’s refined coordination mechanisms, developed through hosting millions during the annual pilgrimage. This planning also leveraged a centralized coordination framework, involving the MoH, GACA, and other government agencies. Sector leaders were well-versed in risk management and preparedness, understanding the scope, capabilities, and resources of each sector, enabling rapid mobilization in response to evolving circumstances (5, 9). Moreover, the country employed standardized tools for hazard prioritization and risk assessment. These tools—designed to evaluate the probability and impact of potential hazards—also helped identify underlying causes of public health emergencies, establish foundational data for research and preparedness planning, and enhance coordination across multiple sectors. Importantly, the system integrated real-time data analytics to support timely and evidence-based responses (10).

As part of its risk management and preparedness strategy, Saudi Arabia employed tools to assess hazard probability and consequences, analyze underlying causes of health emergencies, and generate foundational data to support decision-making and research. These tools also aimed to improve multisectoral coordination and integrate real-time data analytics to guide timely interventions (10). By integrating both national experiences and international best practices, Saudi Arabia was able to develop a robust, flexible, and evidence-based response to the COVID-19 pandemic.

### 2.3 Risk assessment, communication, and mitigation

Risk assessment was central to Saudi Arabia’s approach. A robust, integrated risk management framework was developed to manage Hajj and understand the evolving situation’s impact on global health security, enabling early detection and rapid decision-making. The use of tools such as the “Jeddah Tool” and “Salem COVID Tool” helped guide decisions on travel restrictions, mass gathering cancellations, and the limited Hajj pilgrimage in 1441 AH (2020) (9). The aim was to flatten the epidemic curve, protect the healthcare system, and ensure a low case fatality rate.

Risk communication and community engagement efforts were integrated into all phases of Hajj planning and execution: pre-Hajj, during Hajj, and post-Hajj. This multiphased approach allowed for effective dissemination of accurate information, combating misinformation (the ‘infodemic’), and managing public expectations (11). Saudi Arabia implemented targeted communication strategies across various platforms, which included television, social media, and direct engagement with key communities, ensuring that information reached the most affected groups. Targeted communication strategies, including television, social media, and direct engagement with key communities, ensured information reached the most affected groups. Saudi Arabia adapted media campaigns initially developed for Hajj pilgrims to address the broader population, ensuring that healthcare staff, government officials, and the public were well-informed and prepared to respond effectively to the pandemic.

This integrated approach to risk assessment, communication, and community engagement highlights the synergistic relationship between these elements in Saudi Arabia’s successful COVID-19 response. By identifying risks early, maintaining open communication channels, and engaging the public in the response process, Saudi Arabia was able to implement timely and effective mitigation measures, protect public health, and maintain the continuity of essential religious practices. The coordinated efforts ensured that Saudi Arabia’s pandemic response remained flexible and adapted to new challenges as the situation evolved.

### 2.4 Surveillance, rapid response, and case investigation

The robust surveillance infrastructure developed for monitoring the health of millions of Hajj pilgrims was effectively adapted and utilized during the COVID-19 pandemic. The nation’s experience in case investigation and contact tracing, gained from managing the complexities of mass gatherings, enabled the swift identification, isolation, and tracking of COVID-19 cases (12). In 2019, MoH developed and launched the Health Early Warning System (HEWS), a passive surveillance program triggered by predefined thresholds and connected to electronic health records from various institutions. This program was rolled out nationally soon after the pandemic emerged and, alongside different systems, became pivotal in containing the pandemic (13).

Furthermore, the Kingdom leveraged its experience with mass screening initiatives to enhance its COVID-19 response. In response to the COVID-19 pandemic, Saudi Arabia initiated a large-scale screening program for early detection and prompt containment of the virus. The first phase concentrated on testing individuals in densely populated areas through field assessments, which were executed across 807 locations. The second phase was facilitated by a self-assessment tool categorizing users into low- or high-risk groups. The primary target population for this phase was the low-risk group, who were screened at designated primary healthcare centers. The third phase involved testing asymptomatic individuals suspected of having COVID-19 at specialized drive-through testing centers (5). By the end of 2020, over 20 million tests had been conducted, making Saudi Arabia one of the top countries for per capita testing, demonstrating the Kingdom’s commitment to surveillance and early detection (14).

### 2.5 Border health and points of entry

Managing points of entry is a critical function during Hajj. The stringent measures implemented at borders, informed by the experience of accommodating diverse pilgrims, facilitated effective screening, quarantine, and monitoring of incoming travelers. Before the adoption of a vaccine for COVID-19, the unique efforts made by the Kingdom of Saudi Arabia were distinguished at entry points by the application of precautionary preventive measures in terms of social distancing, face masks, activating clear walking routes to reduce and isolate active cases, and restriction of international flights. Additionally, staff were trained, and logistical arrangements were made to provide enough space, clear walking paths, and adequate equipment at entry points to effectively assess and manage passengers who might be infected or ill, either on board or upon arrival. These measures included training over 5,000 health workers specifically to manage COVID-19 cases at entry points, ensuring effective control at borders. Subsequently, when COVID-19 vaccines became available, preventive measures were eased but continued by updating procedure guidelines and applying entry screening on all travelers arriving from outside at any entry point. These ongoing efforts were vital in ensuring the health and safety of pilgrims and the broader population during Hajj, minimizing the risk of COVID-19 transmission and preventing outbreaks (5). It was crucial to develop and implement public health contingency plans at all points of entry, especially those receiving Hajj and Umrah pilgrims. These plans and the staff trained about them needed to be tested before applying them while ensuring coordination between all relevant entities and constantly updating them.

## 3 Implementation and response

### 3.1 Infection prevention and control

The Ministry of Health (MOH) worked in close collaboration with a range of national and local authorities to implement unique, context-specific measures aimed at mitigating the spread of COVID-19, focusing on enhancing infection prevention and control measures. Saudi Arabia adopted a community response model (CRR and Haddon’s matrix) that was specifically adapted to its public health needs and religious framework (15). This model combined elements of the Haddon matrix, which considers human, agent, and environmental factors, with the CRR tool that emphasizes the Five E’s of prevention (Education, Enforcement, Engineering, Economics, and Emergency response). The integration of these tools was central to structuring the pandemic response in a way that was both systematic and aligned with Saudi Arabia’s unique socio-cultural and religious context, particularly the challenges posed by mass gatherings such as the Hajj.

Efforts to prepare health facilities included the development of procedural guidelines, extensive staff training on infection control principles, and ensuring adequate supplies of personal protective equipment (PPE) and hand sanitizers were available. Laboratories were accredited by the Saudi Center for Disease Prevention and Control (SCDC) to conduct COVID-19 tests, further strengthening facility preparedness. A robust surveillance and recording mechanism was established to closely monitor and investigate confirmed cases among healthcare workers, facilitating prompt decision-making. Guidelines were issued for various societal segments, and educational materials were disseminated to raise awareness about COVID-19 prevention (16).

Health protocols were issued for different sectors, with guidelines tailored to each sector’s specific risks and needs. The issuance of health protocols for different sectors and the contribution of organizations such as the Central Board for Accreditation of Healthcare Institutions (CBAHI) in accrediting healthcare facilities based on infection prevention and control standards were significant achievements. What set Saudi Arabia apart was its emphasis not just on guidelines but also on a system of continuous assessment and adaptation. This included providing real-time data from regions and hospitals, which helped pinpoint vulnerable areas and enabled targeted interventions.

A national plan for managing PPE supplies and implementing real-time inventory tracking was rolled out, and comprehensive assessments of infection control capacity were continuously conducted, demonstrating Saudi Arabia’s adaptability in responding to a rapidly changing situation. Additionally, strict penalties for non-compliance with health protocols, such as mask-wearing and curfew violations, were enacted, which significantly contributed to reducing virus transmission.

These efforts, supported by advanced surveillance mechanisms and the constant updating of infection control procedures, exemplified a proactive, adaptive response to COVID-19 that was more dynamic than the generalized approaches followed by other nations. Overall, these efforts emphasize a comprehensive approach to infection prevention and control, emphasizing collaboration, preparedness, and surveillance to mitigate the spread of COVID-19.

### 3.2 Case management and knowledge sharing

The MoH’s experience in managing surge capacity and operating seasonal hospitals facilitated its pandemic response in repurposing existing facilities and efficiently allocating resources, showcasing a well-prepared and adaptable healthcare system. Healthcare facilities gathered field and hybrid simulation training in detecting and managing patients and evaluating surge capacity plans and essential services to alleviate pressure in preparation for significant COVID-19 cases. Managing uncertainties and generating best practices with limited knowledge enabled a confident response during the pandemic. Saudi Arabia’s response was significantly shaped by knowledge-sharing practices developed through years of managing mass gatherings such as the Hajj. This institutional culture of continuous improvement enabled healthcare workers to quickly adapt to evolving clinical guidelines, leading to the rapid adoption of innovative treatment protocols.

During the pandemic, real-time data from hospitals and healthcare workers informed the development of these protocols. This allowed for faster incorporation of the latest research and best practices into the country’s response, particularly in managing severe cases and minimizing transmission. The ability to quickly exchange insights and practical solutions across healthcare networks was critical, distinguishing Saudi Arabia from other countries where knowledge-sharing efforts were often slower or more fragmented.

These measures, based on prior experience with surge capacity and knowledge management, enabled Saudi Arabia to manage its COVID-19 response more effectively than many other nations, particularly those without the infrastructure and expertise derived from handling large numbers of international travelers each year (12).

### 3.3 COVID-19 vaccinations

Saudi Arabia’s ability to execute large-scale health interventions, developed through decades of experience with mass gatherings, was essential in facilitating the rapid rollout of COVID-19 vaccinations. The Kingdom’s established infrastructure for managing large crowds, particularly during Hajj, was adapted to support the distribution and administration of vaccines across the country. This logistical expertise allowed for a smooth and efficient vaccination campaign, ensuring broad coverage and quick access to vaccines. The Saudi Food and Drug Authority (SFDA) authorized the first COVID-19 vaccine as soon as the Phase III clinical trial findings were published, ensuring swift approval and distribution. This agile regulatory response enabled the Kingdom to avoid delays experienced by other nations that struggled with regulatory bottlenecks. The rapid deployment of vaccines was supported by the Kingdom’s digital health transformation, which enabled real-time tracking of immunization status. Vaccination records were linked to digital health platforms, allowing authorities to verify immunization status and track vaccination coverage in real-time. This digital infrastructure was crucial for ensuring that individuals had access to vaccines and enforcing policies that required proof of vaccination for entry into public and government facilities. The recent digital health transformation in Saudi Arabia presents a distinct opportunity to accelerate drug surveillance and verify immunization status before accessing public and government facilities (17, 18). The Kingdom’s proactive approach in securing adequate vaccine supplies and implementing a comprehensive vaccination distribution plan ensured that every citizen and resident had access to the vaccine, contributing to the country’s goal of achieving herd immunity and curbing the spread of the virus (17). Saudi Arabia’s mass-gathering infrastructure played a crucial role in enabling swift vaccination distribution, distinguishing its vaccination rollout from other countries that did not have similar large-scale logistics capabilities.

### 3.4 Considering vulnerable and marginalized populations

Engaging diverse international pilgrims provided MoH with informed direction and resources to reach all expatriates early in the pandemic. The nation considered vulnerable and marginalized populations, ensuring equitable access to healthcare services and vaccination campaigns. This approach reflected a commitment to addressing health disparities, drawing from the principles of inclusivity ingrained in mass gathering preparations.

Saudi Arabia’s ability to navigate the legal and financial dimensions of the pandemic was informed by the experiences gained from organizing the Hajj. The legislative frameworks and financing mechanisms established for mass gatherings seamlessly translated into a comprehensive response to COVID-19. The ability to swiftly allocate resources and enact legislation demonstrated the adaptability and foresight ingrained in Saudi Arabia’s health governance structures. Notably, the Saudi government provided free medical care to all individuals, regardless of their legal residency status.

### 3.5 Public health and social measures

The strategic deployment of public health and social measures during the Hajj pilgrimage demonstrated a keen understanding of population dynamics gleaned from years of experience in managing mass gatherings. The implementation of social distancing, mask mandates, and crowd control strategies was adapted to the unique context of the Hajj, ensuring their feasibility and effectiveness within the densely populated environment of the holy sites. Extensive campaigns promoting hand hygiene and respiratory etiquette were implemented, emphasizing their importance in preventing the spread of COVID-19 among pilgrims.

Hand sanitizing stations were strategically placed throughout the pilgrimage areas, and regular disinfection and cleaning of frequently touched surfaces and objects were prioritized. Screening and testing protocols for pilgrims were regularly updated based on the evolving pandemic situation and scientific evidence, ensuring early detection and containment of potential cases. These protocols included pre-Hajj testing, symptom checks, and targeted testing for individuals with potential exposure. The successful implementation of these measures during Hajj underscored the Kingdom’s ability to leverage its expertise in managing large-scale events to address the public health challenges posed by the COVID-19 pandemic.

### 3.6 Mental health and psychosocial support

Recognizing the mental health impact of the pandemic, Saudi Arabia leveraged its experience in managing the emotional wellbeing of pilgrims during Hajj. Psychosocial support strategies, including counseling services and community engagement initiatives, were integrated into the pandemic response, addressing the holistic needs of the population.

As part of its response to the pandemic, Saudi Arabia’s mental health support efforts were uniquely informed by its previous management of pilgrims’ wellbeing during Hajj, and these strategies were adapted and expanded to include telehealth services for psychological support.

## 4 Outcomes and lessons learned

Saudi Arabia’s proactive response to the COVID-19 pandemic, particularly its successful management of the Hajj and Umrah pilgrimages, yielded measurable outcomes and evidence-based insights for addressing future public health crises. The measures implemented resulted in the effective containment of COVID-19 among pilgrims, with zero reported cases during the 2020 Hajj, serving as a model for managing large-scale events (19). This success is a direct outcome of stringent preventive measures and careful policy implementation, showcasing the effectiveness of the intervention. By prioritizing both public health safety and the continuation of religious rituals, the Kingdom achieved notable success in containing the spread of COVID-19 among pilgrims. This success was further emphasized by the Kingdom’s case fatality rate remaining below the global average and a recovery rate exceeding 90% during peak periods, reflecting the effectiveness of its experience-driven response strategy (20).

These outcomes emphasize the effectiveness of Saudi Arabia’s experience-driven, adaptive response strategy. Additionally, the Kingdom’s ability to maintain essential services while enforcing health measures—such as restrictions on gatherings and enhanced border control protocols—was a key achievement. This success became a model for managing large-scale events, demonstrating the critical role of centralized coordination and data-driven decision-making in crisis management. The integration of digital innovations, such as health-tracking apps and contact tracing systems, was pivotal in real-time surveillance and response, underlining the intervention’s unique features.

By the end of 2020, over 20 million COVID-19 tests had been conducted, placing Saudi Arabia among the top countries globally in per capita testing. These outcomes demonstrate the Kingdom’s robust surveillance, early detection, and containment efforts. Additionally, the Kingdom’s ability to maintain essential services while enforcing health measures—such as restrictions on gatherings and enhanced border control protocols—was a key achievement. This success became a model for managing large-scale events, illustrating how centralized coordination and data-driven decision-making can enhance national resilience.

Saudi Arabia’s experience during the COVID-19 pandemic highlighted several key lessons. The importance of clear, consistent communication across diverse platforms and the critical role of early warning systems, mass screening, and contact tracing were clearly demonstrated through policy implementation outcomes. The Kingdom also recognized the need to prioritize vulnerable populations, implement basic public health measures, and ensure efficient logistics and supply chain management. The pandemic further emphasized the value of supporting healthcare workers’ wellbeing and mental health.

Finally, Saudi Arabia’s response demonstrated the potential of digital innovation in public health. The successful integration of digital tools such as Sehhaty and Tawakkalna played a crucial role in managing the pandemic. These apps supported the enforcement of health measures, managing movement permits, and monitoring of COVID-19 cases in real-time. However, their rapid deployment also introduced cybersecurity challenges, highlighting the need for strong cybersecurity frameworks and user training to protect sensitive data. This experience underscored the dual nature of digital tools—offering essential public health benefits while requiring ongoing risk mitigation strategies to ensure safety, trust, and sustained use (21).

## 5 Conclusion

Following the end COVID-19 pandemic, Saudi Arabia’s outstanding response is a testament to the enduring benefits of its experience and capacity building from organizing the annual Hajj pilgrimage. By operationalizing the WHO After-Action Review pillars, the Kingdom translated mass-gathering insights into concrete measures. Multisectoral command structures, real-time data analytics, targeted communication campaigns, and scalable surge-capacity plans together formed a cohesive, legacy-driven resilience strategy. As the world readies for future health crises, Saudi Arabia’s model showcases how leveraging cultural and organizational assets can yield agile, effective public-health responses.

Embedding these pillars within national preparedness frameworks will sustain and expand the Kingdom’s pandemic capabilities. By leveraging existing mass-gathering infrastructures and institutional knowledge, countries can build resilient, adaptive systems capable of meeting the challenges of tomorrow’s public-health crises.

## Data Availability

The original contributions presented in the study are included in the article/supplementary material, further inquiries can be directed to the corresponding author.
